# Association between hemoglobin-to-red blood cell distribution width ratio and hospital mortality in patients with non-traumatic subarachnoid hemorrhage

**DOI:** 10.3389/fneur.2023.1180912

**Published:** 2023-06-14

**Authors:** Jiuling Liu, Junhong Wang

**Affiliations:** ^1^Department of Neurology, Nanjing BenQ Medical Center, The Affiliated BenQ Hospital of Nanjing Medical University, Nanjing, China; ^2^Department of Neurosurgery, Tongji Hospital, Tongji Medical College, Huazhong University of Science and Technology, Wuhan, Hubei, China

**Keywords:** hospital mortality, hemoglobin-to-red blood cell distribution width ratio, non-traumatic subarachnoid hemorrhage, intensive care unit, MIMIC-IV

## Abstract

**Background:**

In patients with ischemic stroke, low hemoglobin-to-red blood cell distribution width ratio (HRR) was associated with an increased risk of mortality. However, it was unknown in the non-traumatic subarachnoid hemorrhage (SAH) population. The purpose of this study was to examine the association between baseline HRR and in-hospital mortality in patients with non-traumatic SAH.

**Methods:**

Non-traumatic SAH patients were screened out of the Medical Information Mart for Intensive IV (MIMIC-IV) database between 2008 and 2019. The Cox proportional hazard regression models were utilized to analyze the association between baseline HRR and in-hospital mortality. Restricted cubic splines (RCS) analysis was utilized to determine the relationship curve between hospital mortality and the HRR level and examine the threshold saturation effect. We further applied Kaplan–Meier survival curve analysis to examine the consistency of these correlations. The interaction test was used to identify subgroups with differences.

**Results:**

A total of 842 patients were included in this retrospective cohort study. Compared with individuals with lower HRR Q1 ( ≤ 7.85), the adjusted HR values in Q2 (7.86–9.15), Q3 (9.16–10.16), and Q4 (≥10.17) were 0.574 (95% CI: 0.368–0.896, *p* = 0.015), 0.555 (95% CI: 0.346–0.890, *p* = 0.016), and 0.625 (95% CI: 0.394–0.991, *p* = 0.045), respectively. The association between the HRR level and in-hospital mortality exhibited a non-linear relationship (*p* < 0.05). The threshold inflection point value of 9.50 was calculated using RCS analysis. When the HHR level was lower than 9.50, the risk of in-hospital mortality rate decreased with an adjusted HR of 0.79 (95% CI: 0.70–0.90, *p* = 0.0003). When the HRR level was higher than 9.50, the risk of in-hospital mortality almost hardly increased with the increase in the HRR level (adjusted HR = 1.18, 95% CI: 0.91–1.53, *p* = 0.2158). K-M analysis showed that patients with low HRR levels had significantly higher in-hospital mortality (*p* < 0.001).

**Conclusion:**

There was a non-linear connection between the baseline HRR level and in-hospital mortality. A low level of HRR could increase the risk of death in participants with non-traumatic SAH.

## Introduction

Subarachnoid hemorrhage (SAH) is a common form of stroke in the intensive care unit (ICU) and a potentially devastating illness (1). It affects nearly 10 percent of every 1,00,000 individuals and accounts for nearly 5% of all strokes each year (2, 3). However, long-term disability and mortality from SAH accounted for 27% of all stroke-related potential years of life lost before the age of 65 years (4). Although the optimal management of SAH has improved, hospital mortality and severe disability are still high (5).

Inflammatory reactions play an important role during the early brain injury induced by SAH and significantly affect the outcomes (6). Experiments have suggested that the mechanism leading to this situation may be extravasated red blood cells in the subarachnoid space undergoing degradation, releasing a host of bioactive and pro-inflammatory properties including hemoglobin (Hb), methemoglobin, and bilirubin (7, 8). Vasoactive factors and inflammation are released, exacerbating brain edema, oxidative stress damage, and cell apoptosis, leading to disruption of the blood–brain barrier (9, 10). Hemoglobin reflects the patient's degree of anemia, and red-blood-cell distribution width (RDW) reflects the heterogeneity in the sizes of erythrocytes (11, 12). In the inflammatory state, the lifespan of red blood cells is shortened, leading to anemia and an increase in RDW.

Previous studies have shown that RDW was a parameter reflecting inflammation (13, 14) and related to the outcomes of SAH patients (15–19). In addition, Hb is an important component of the complete blood count and related to nutritional status (20) and immune response (21). However, the level of RDW and Hb may be affected by many factors, such as medications, nutritional status, oxidative stress, and blood transfusion (13, 22–24). Therefore, Hb/RDW (HRR) is a relatively good parameter for reducing the impact of the factors mentioned. HRR is an easily obtained parameter. Previous investigations have shown that HRR was associated with inflammation (25–27). In recent years, more and more evidence has shown that low level of HRR was closely related to significantly deteriorating prognosis in many critically ill patients, such as coronary heart disease, sepsis, and ischemic stroke (28–30). However, the relationship between HRR and mortality in patients with non-traumatic SAH is lacking.

In this study, we aim to test the association between baseline HRR and hospital mortality among critically ill patients with non-traumatic SAH.

## Materials and methods

### Data sources

The study data were downloaded freely from a large publicly accessed database called Medical Information Mart for Intensive Care (MIMIC-IV) (31). This database contains information on patients admitted to the Beth Israel Deaconess Medical Center (BIDMC) between 2008 and 2019. Posterior to the completion of the National Institutes of Health (NIH) training course and the Protecting Human Research Participants test, one researcher Junhong Wang obtained approval to exploit the database (Record ID, 45677587). The study was carried out following the Helsinki Declaration guidelines and was reviewed and approved by the Massachusetts Institute of Technology and the Institutional Review Board of BIDMC. All data were anonymous to protect patient privacy, and the need for informed consent was waived. This study follows the Strengthening the Reporting of Observational Studies in Epidemiology (STROBE) statement (32).

### Study population

**Figure 1:**
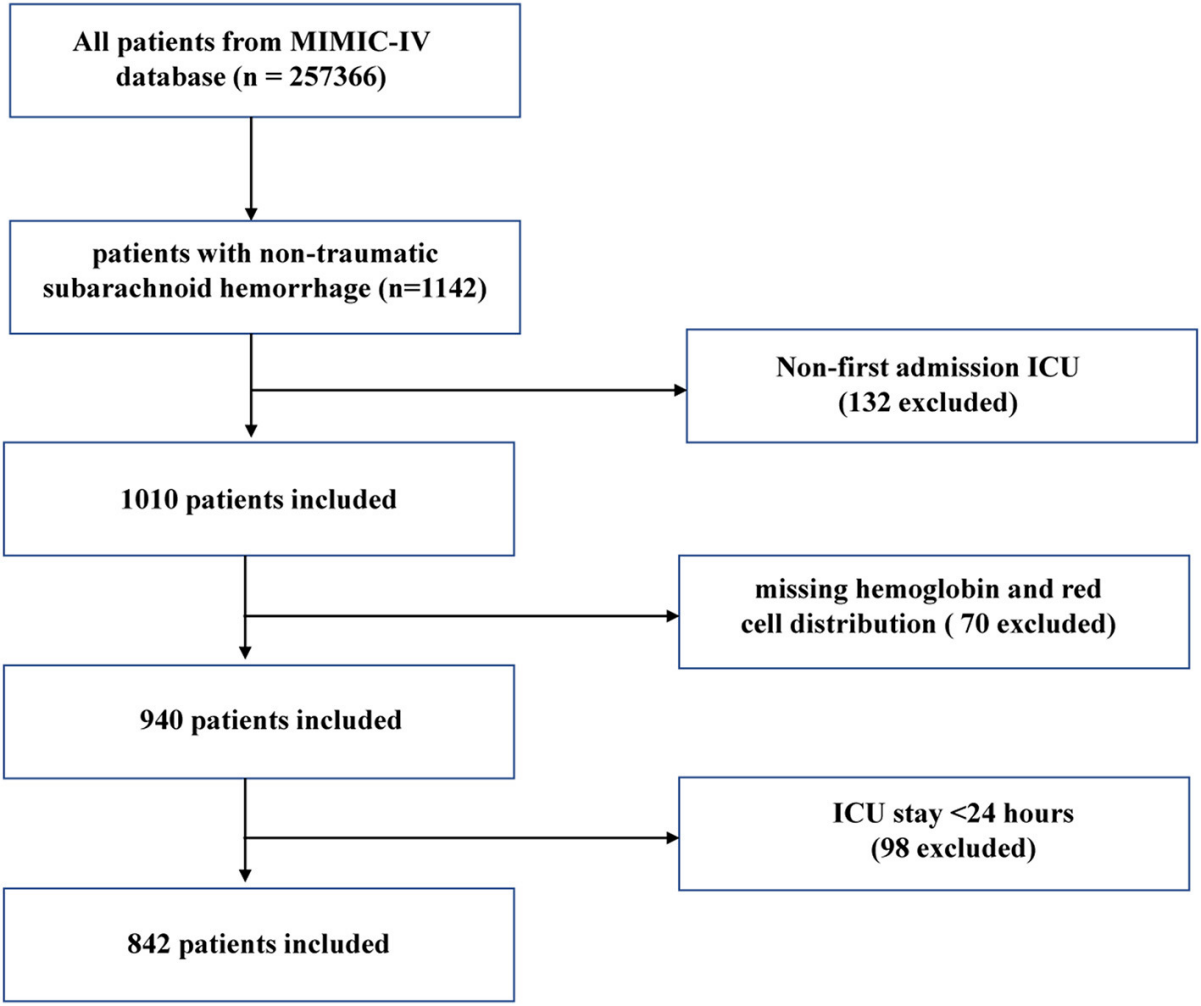
Flow chart of the study. MIMC-IV, Medical Information Mart for Intensive Care IV; ICU, intensive care unit.

A total of 1,142 patients with non-traumatic SAH were selected based on the record of ICD-9 code 430 and ICD-10 code I60. Patients who met the requirements were selected to undergo analysis: (1) first ICU admission; (2) age > 18 years. The exclusion criteria were as follows: (1) ICU patients with a length of stay < 24 h and (2) participants who had missing hemoglobin or red blood cell distribution width value. Finally, 842 patients were included in this study (Figure 1).

### Data extraction

Structured Query Language (SQL) and PostgreSQL are used to extract the following variables from the MIMIC-IV database in our study: (1) demographic variables include sex, age, and ethnicity. (2) vital signs include the heart rate, respiratory rate (RR), mean blood pressure (MBP), systolic blood pressure (SBP), diastolic blood pressure (DBP), percutaneous oxygen saturation (SpO_2_), and temperature. (3) Comorbidities include myocardial infarction, congestive heart failure, chronic pulmonary disease, paraplegia, hypertension, diabetes, and sepsis. (4) Laboratory variables include glucose, platelet, white blood cell (WBC) count, calcium, mean corpuscular hemoglobin (MCH), prothrombin time (PT), activated partial thromboplastin time (APTT), Hb, and RDW. The first test of RDW and Hb were included within 24 h after entering the ICU. (5) Severity at admission was identified *via* the Glasgow coma score (GCS) and World Federation of Neurosurgical Societies (WFNS). (6) Delayed cerebral ischemia (DCI), hydrocephalus, Length of ICU stay, length of the hospital, and in-hospital mortality. (7) HRR was calculated using the following formula: HRR = Hb (g/L)/RDW (%) (29, 30, 33).

### Endpoints

The outcome was in-hospital mortality, which was described by the patient's survival status at the moment of discharge from the hospital.

### Statistical analysis

The continuous variates were displayed as average ± standard deviation (SD) or mid-value (interquartile range). The Student's *t*-test or Mann–Whitney *U*-test was used according to the normality of the distribution. Categorical variates were displayed as case quantity (%), and the chi-square test (or Fisher's exact approach) was utilized for analyses of the difference between the different HRRs (four quantiles) (29).

To reduce the interference of potential confounding factors on in-hospital mortality, univariate and multivariate Cox proportional hazard regression analyses were performed. The screening of confounders was based on the following criteria: (1) The factor had a significant impact (>10%) on the research variate. (2) Certain factors may have a significant impact on the outcome variate based on previous experiences. (3) For univariate analysis, our team modified the variates (*p* < 0.05). In the multivariate case, we performed several statistical models to ensure that the results were stable. In the crude model, no variables were adjusted. In model I, age, sex, and ethnicity variables were adjusted, while model II further adjusted other 16 variables, including heart rate, RR, WBC, PT, APTT, congestive heart failure, Charlson comorbidity index, endovascular therapy, sepsis, norepinephrine, vasopressin, ventilation, GCS, WFNS, DCI, and hydrocephalus.

Restricted cubic spline analysis was employed to determine whether there was a curve relationship between hospital mortality. If the non-linear correlation was observed, a two-piecewise linear regression model was performed to calculate the threshold effect of the HRR on hospital mortality in terms of the smoothing plot (34). The turning point for the HRR was determined using “exploratory” analyses, which is to move the trial turning point along the pre-defined interval and pick up the one which gave maximum model likelihood. We also performed a log-likelihood ratio test and compared the one-line linear regression model with the two-piece-wise linear model (35). We used the bootstrap resampling method to calculate the 95% CI for the turning point, as described in the previous analysis (30, 34, 36).

Furthermore, interactions and stratified analyses were conducted using age (< 65 and ≥65 years old), sex, WBC counts, myocardial infarction, congestive heart failure, chronic pulmonary disease, renal disease, paraplegia, hypertension, sepsis, endovascular therapy of aneurysm, and GCS (< 8 and ≥8), as previously described. Missing values were filled with mean values (37). The details of the missing values are shown in Supplementary Table 1.

A two-tailed *p*-value of < 0.05 was considered to be statistically significant in all analyses. All statistical analyses were carried out using EmpowerStats (http://www.empowerstats.com, version 3.6.1 R software package) software (35).

## Results

### Baseline characteristics of the study patients

After the screening, 1,010 patients were admitted to the ICU due to non-traumatic SAH for the first time, of whom 842 were selected for the final data analysis (see the flowchart in Figure 1). The distribution of the baseline characteristics of the population according to baseline HRR levels in quartiles is described in Table 1 (Q1: ≤ 7.85, Q2: 7.86–9.15, Q3: 9.16–10.16, and Q4: ≥10.17). The mean age was 61.2 ± 14.9 years, and ~55.9% of these were women. The demographics, vital signs, comorbidities, laboratory variables and scoring, and other related data according to HRR levels are presented in Table 1. According to Table 1, we found that there were significant differences in age, sex, ethnicity, heart rate, DBP, MBP, RR, congestive heart failure, renal disease, sepsis, WBC, PT, APTT, GCS, and WFNS. Compared with patients in the Q1 group, patients with HRR in Q2, Q3, and Q4 groups were at lower risk of in-hospital mortality and ICU mortality.

**Table 1:** Population characteristics by quartiles of the baseline HRR level.

**Variables**	**Quartiles of HRR**					***p-*value**
	**Total (*n* = 842)**	**Q1 ( ≤ 7.85) (*n* = 211)**	**Q2 (7.86–9.15) (*n* = 209)**	**Q3 (9.16–10.16) (*n* = 211)**	**Q4 (≥10.17) (*n* = 211)**	
**Demographic**						
Female, *n* (%)	471 (55.9)	136 (64.5)	151 (72.2)	111 (52.6)	73 (34.6)	< 0.001
Age, years	61.2 ± 14.9	64.4 ± 15.2	63.3 ± 16.0	59.8 ± 13.5	57.3 ± 13.5	< 0.001
Ethnicity, *n* (%)						0.022
Asian	32 (3.8)	8 (3.8)	9 (4.3)	8 (3.8)	7 (3.3)	
White	507 (60.2)	109 (51.7)	128 (61.2)	135 (64)	135 (64)	
Black	68 (8.1)	30 (14.2)	16 (7.7)	13 (6.2)	9 (4.3)	
Other	235 (27.9)	64 (30.3)	56 (26.8)	55 (26.1)	60 (28.4)	
**Vital signs**						
Heart rate, beats/min	78.7 ± 13.1	81.5 ± 14.3	77.3 ± 12.4	77.2 ± 12.4	78.7 ± 12.7	< 0.001
SBP, mmHg	125.3 ± 12.9	123.4 ± 14.3	126.1 ± 12.6	125.2 ± 13.6	126.5 ± 10.8	0.069
DBP, mmHg	64.3 ± 8.9	61.7 ± 9.2	62.2 ± 8.6	65.7 ± 7.7	67.5 ± 8.7	< 0.001
MBP, mmHg	82.5 ± 8.6	80.2 ± 8.9	81.9 ± 8.6	83.9 ± 8.3	84.1 ± 8.0	< 0.001
RR, times/min	18.2 ± 3.2	18.9 ± 3.5	17.9 ± 2.9	17.7 ± 3.1	18.0 ± 3.2	< 0.001
Temperature, °C	37.0 ± 0.5	37.0 ± 0.7	37.0 ± 0.4	37.0 (36.8, 37.3)	37.0 (36.8, 37.3)	0.383
SpO_2_, %	97.5 ± 1.9	97.8 ± 2.0	97.8 ± 1.8	97.2 ± 2.1	97.2 ± 1.7	< 0.001
**Comorbidities**, ***n*** **(%)**						
Myocardial infarction	61 (7.2)	21 (10)	17 (8.1)	11 (5.2)	12 (5.7)	0.203
Congestive heart failure	70 (8.3)	34 (16.1)	13 (6.2)	16 (7.6)	7 (3.3)	< 0.001
Chronic pulmonary disease	122 (14.5)	38 (18)	36 (17.2)	26 (12.3)	22 (10.4)	0.072
Paraplegia	139 (16.5)	44 (20.9)	38 (18.2)	32 (15.2)	25 (11.8)	0.075
Renal disease	54 (6.4)	35 (16.6)	9 (4.3)	5 (2.4)	5 (2.4)	< 0.001
Hypertension	423 (50.2)	105 (49.8)	117 (56)	101 (47.9)	100 (47.4)	0.269
Diabetes	39 (4.6)	15 (7.1)	10 (4.8)	9 (4.3)	5 (2.4)	0.142
Sepsis	424 (50.4)	127 (60.2)	116 (55.5)	98 (46.4)	83 (39.3)	< 0.001
Charlson comorbidity index	4.0 (3.0, 6.0)	5.0 (4.0, 8.0)	5.0 (3.0, 6.0)	4.0 (3.0, 5.0)	4.0 (3.0, 5.0)	< 0.001
**Laboratory results**						
Platelets, 10^9^/L	230.0 (187.0, 280.8)	219.0 (161.5, 284.5)	238.0 (188.0, 279.0)	232.0 (191.5, 276.0)	231.0 (193.5, 281.5)	0.186
WBC, 10^9^/L	12.9 (9.9, 16.5)	12.9 (8.9, 17.2)	12.5 (10.0, 15.9)	12.7 (10.0, 15.3)	14.0 (10.8, 17.3)	0.046
Hemoglobin, g/L	12.2 ± 1.9	9.9 ± 1.4	11.7 ± 0.8	12.8 ± 0.7	14.2 ± 1.1	< 0.001
RDW, %	13.9 ± 1.6	15.6 ± 2.1	13.6 ± 0.8	13.3 ± 0.7	12.9 ± 0.8	< 0.001
Glucose, mg/dl	130.9 (113.4, 153.5)	128.6 (110.6, 158.7)	135.6 (114.0, 150.8)	129.0 (113.2, 151.0)	131.8 (114.6, 154.0)	0.756
Calcium, mg/dl	8.7 (8.3, 9.1)	8.6 (8.2, 9.1)	8.7 (8.3, 9.1)	8.7 (8.4, 9.0)	8.9 (8.6, 9.2)	< 0.001
MCH, pg	30.5 (29.2, 31.8)	29.5 (27.1, 30.9)	30.4 (29.3, 31.6)	30.7 (29.6, 31.8)	31.2 (29.9, 32.4)	< 0.001
PT, s	12.6 (11.7, 13.8)	13.6 (12.2, 15.8)	12.5 (11.8, 13.4)	12.3 (11.5, 13.1)	12.4 (11.6, 13.4)	< 0.001
APTT, s	28.8 (25.9, 33.1)	29.6 (26.9, 36.7)	28.4 (25.6, 32.8)	27.8 (25.6, 31.6)	29.1 (26.4, 32.0)	0.003
**Therapy**, ***n*** **(%)**						
Norepinephrine	30 (3.6)	17 (8.1)	5 (2.4)	4 (1.9)	4 (1.9)	< 0.001
Vasopressin	8 (1.0)	4 (1.9)	0 (0)	1 (0.5)	3 (1.4)	0.193
Ventilation	429 (51.0)	130 (61.6)	111 (53.1)	95 (45)	93 (44.1)	< 0.001
Clipping of aneurysm	35 (4.2)	6 (2.8)	10 (4.8)	13 (6.2)	6 (2.8)	0.410
Endovascular therapy of aneurysm	201 (23.9)	44 (20.9)	60 (28.7)	53 (25.1)	44 (20.9)	0.050
**Scores**						
GCS	12.0 (7.0, 14.0)	10.0 (7.0, 14.0)	10.0 (6.0, 14.0)	13.0 (8.0, 14.0)	13.0 (7.5, 14.0)	< 0.001
WFNS						0.003
I	127 (15.1)	24 (11.4)	27 (12.9)	39 (18.5)	37 (17.5)	
II	172 (20.4)	32 (15.2)	33 (15.8)	50 (23.7)	57 (27)	
III	115 (13.7)	29 (13.7)	26 (12.4)	34 (16.1)	26 (12.3)	
IV	185 (22.0)	51 (24.2)	57 (27.3)	39 (18.5)	38 (18)	
V	243 (28.9)	75 (35.5)	66 (31.6)	49 (23.2)	53 (25.1)	
**Outcomes**						
Delayed cerebral ischemia	64 (7.60%)	7 (3.32%)	14 (6.70%)	19 (9.00%)	24 (11.37%)	0.014
Hydrocephalus	77(9.14)	19 (9.00%)	18 (8.61%)	16 (7.58%)	24 (11.37%)	0.581
Length of ICU stay, days	7.0 (2.9, 12.9)	5.9 (2.7, 11.9)	7.5 (2.9, 13.8)	6.8 (3.2, 13.8)	7.1 (2.6, 12.7)	0.408
Length of hospital stay, days	11.3 (6.7, 18.7)	11.8 (6.0, 19.9)	12.1 (7.4, 19.3)	10.8 (6.2, 17.1)	10.5 (6.7, 16.4)	0.275
In-hospital mortality	166 (19.7)	71 (33.6)	35 (16.7)	29 (13.7)	31 (14.7)	< 0.001

SBP, systolic blood pressure; DBP, diastolic blood pressure; MBP, mean blood pressure; RR, respiratory rate; SpO_2_, percutaneous oxygen saturation; HRR, hemoglobin/red cell distribution width; WBC, white blood cell; MCH, mean corpuscular hemoglobin; PT, prothrombin time; APTT, activated partial thromboplastin time; GCS, Glasgow coma score; WFNS, World Federation of Neurosurgical Societies; ICU, Intensive care unit; SAH, subarachnoid hemorrhage.

The non-survivor group presented lower HRR (median: 8.5 vs. 9.3, *p* < 0.001). Compared with the survivor group, the non-survivor group was older (66.8 ± 14.9 vs. 59.8 ± 14.5 years old, *p* < 0.001) and presented a higher comorbidity incidence, such as congestive heart failure, sepsis, chronic pulmonary disease, Charlson comorbidity index, as well as lower GCS scores (all *p*-values < 0.05) (Supplementary Table 2).

### The association between baseline HRR and in-hospital mortality

**Figure 2:**
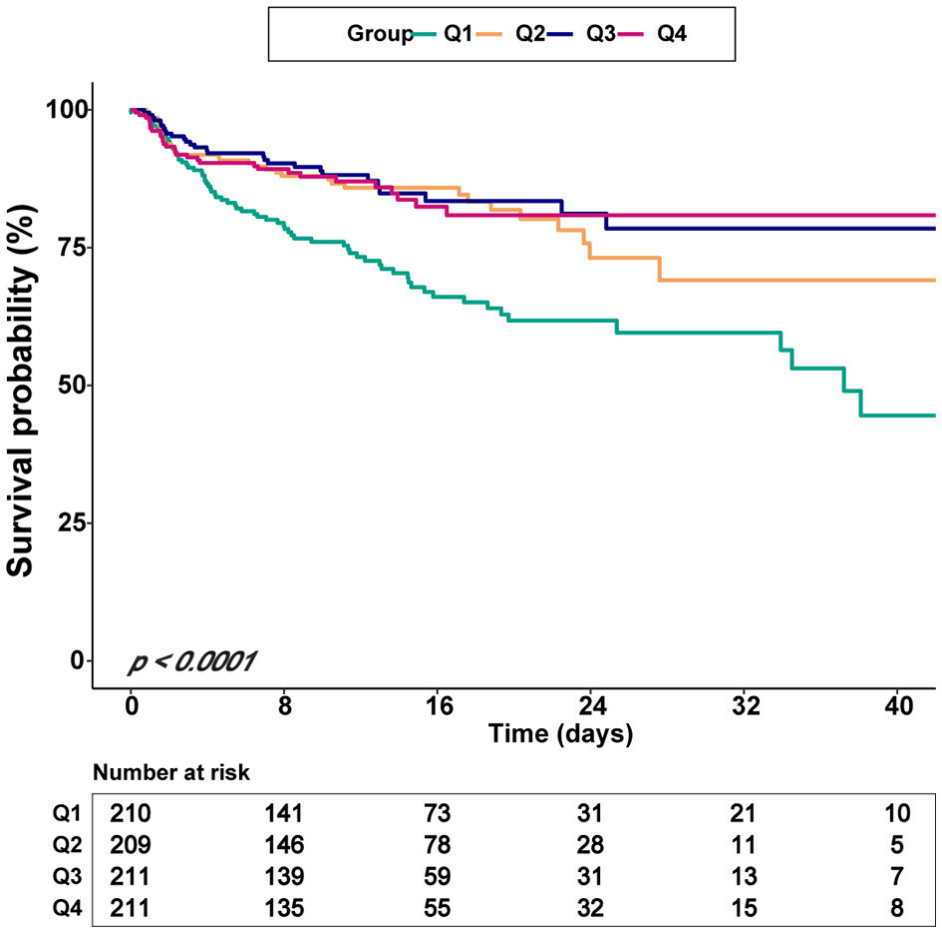
Kaplan–Meier survival curves for critically ill patients with non-traumatic SAH based on the baseline HRR level. X-Axis: survival time (days). Y-Axis: survival probability. HRR, hemoglobin/red cell distribution width; SAH, subarachnoid hemorrhage.

The univariate analysis demonstrated that age, heart rate, RR, WBC, PT, APTT, sepsis, Charlson comorbidity index, norepinephrine, vasopressin, ventilation, GCS, WFNS were associated with in-hospital mortality (Supplementary Table 3). Table 2 showed an unadjusted and a multivariable-adjusted association between HRR and in-hospital mortality. In model I, age, sex, and ethnicity variables were adjusted, while model II further adjusted other 16 variables, including heart rate, RR, WBC, PT, APTT, congestive heart failure, Charlson comorbidity index, endovascular therapy, sepsis, norepinephrine, vasopressin, ventilation, GCS, WFNS, DCI, hydrocephalus. When HRR was used as a continuous variable, the results showed that HRR was associated with in-hospital mortality (non-adjusted model: HR = 0.861, 95% CI: 0.798–0.929, *p* = 0.0001; Model I: HR = 0.895, 95% CI: 0.828–0.968, *p* = 0.0052; Model II: HR = 0.887, 95% CI: 0.797–0.964, *p* = 0.007). The in-hospital mortality of non-traumatic SAH decreased with a 1-unit increase in HRR. Moreover, as a classification variable, patients with lower HRR levels had significantly increased in-hospital mortality. Compared to the reference group (Q1 ≤ 7.85), the adjusted HR values for individuals in Q2 (7.86–9.15), Q3 (9.16–10.16), and Q4 (≥10.17) were 0.574 (95% CI: 0.368–0.896, *p* = 0.015), 0.555 (95% CI: 0.346–0.890, *p* = 0.016), and 0.625 (95% CI: 0.394–0.991, *p* = 0.045), respectively (*p* for trend = 0.015). Regarding sensitivity analysis, HRR levels were assessed as a continuous and categorical variable, respectively, with in-hospital mortality, yielding consistent results. In addition, the K-M curves contrasting the four groups were displayed in Figure 2. The figure indicated that the survival rate of group Q1 was lower than groups Q2, Q3, and Q4 (*p* < 0.0001).

**Table 2:** Multivariate cox regression analyses for in-hospital mortality in non-traumatic subarachnoid hemorrhage patients.

**Exposure**	**Non-adjust model**		**Model I**		**Model II**	
	**HR (95% CI)**	***p*-value**	**HR (95% CI)**	***p*-value**	**HR (95% CI)**	***p*-value**
**HRR quartiles**						
Q1 ( ≤ 7.85)	1 (Ref)		1 (Ref)		1 (Ref)	
Q2 (7.86–9.15)	0.500 (0.333, 0.751)	< 0.001	0.522 (0.347, 0.786)	0.002	0.574 (0.368, 0.896)	0.015
Q3 (9.16–10.16)	0.435 (0.282, 0.671)	< 0.001	0.489 (0.315, 0.759)	0.001	0.555 (0.346, 0.890)	0.015
Q4 (≥10.17)	0.447 (0.292, 0.686)	< 0.001	0.534 (0.342, 0.834)	0.006	0.625 (0.394, 0.991)	0.045
*p* for trend	0.742 (0.643, 0.855)	< 0.001	0.784 (0.676, 0.909)	0.001	0.872 (0.780, 0.974)	0.015
HRR (per 1 increases)	0.861 (0.798, 0.929)	0.001	0.895 (0.828, 0.968)	0.005	0.877 (0.797, 0.964)	0.007

Non-adjusted: no covariates were adjusted.

Model I: adjusted for age, sex, and ethnicity.

Model II: adjusted for age, sex, ethnicity, heart rate, RR, WBC, PT, APTT, congestive heart failure, Charlson comorbidity index, endovascular therapy, sepsis, Norepinephrine, vasopressin, ventilation, GCS, WFNS, delayed cerebral ischemia, hydrocephalus.

RR, respiratory rate; WBC, white blood cell; INR, international normalized ratio; PT, prothrombin time; APTT, activated partial thromboplastin time; GCS, Glasgow coma score; WFNS, World Federation of Neurosurgical Societies; HRR, hemoglobin/red cell distribution width; HR, hazard ratio; CI, confidence interval; Ref, reference.

### Analysis of the non-linear relationship between the baseline HRR and in-hospital mortalit*y*

**Figure 3:**
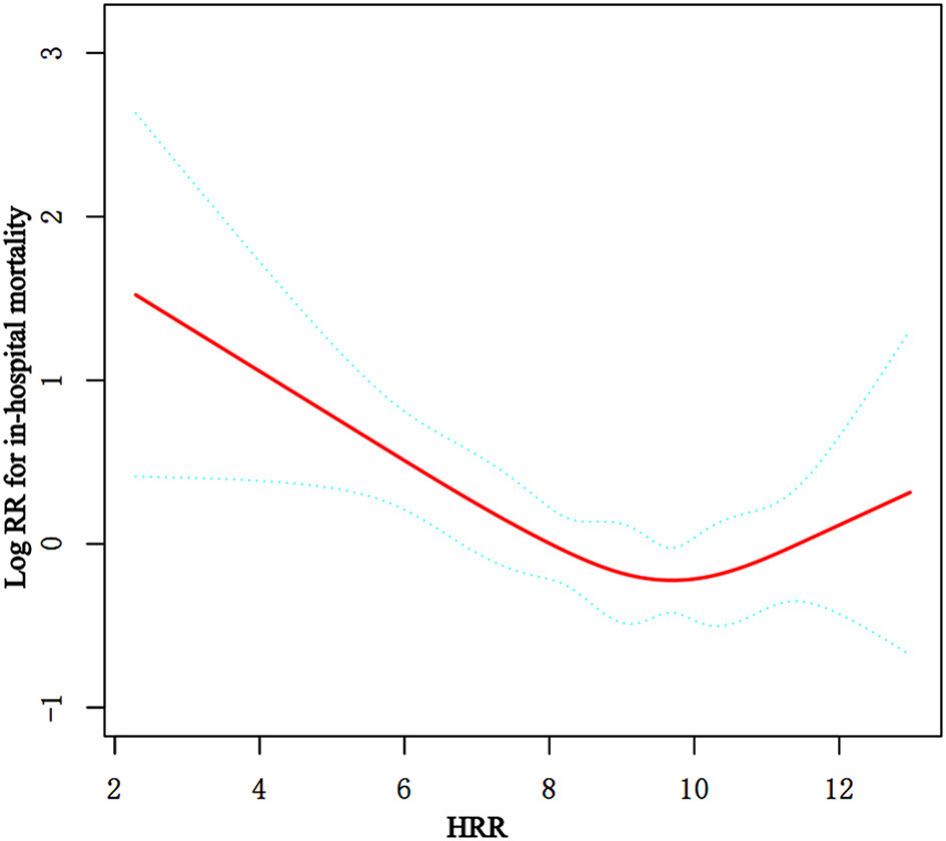
Non-linear relationship observed between in-hospital mortality and the baseline HRR level, and the slope changes evidently, which may have a threshold saturation effect. The solid red line represents the smooth curve fit between variables. Blue bands present the 95% confidence interval. The data were adjusted for the variables in Model II in Table 2.

After adjusting the variables in Model II, a curve-fitting equation for baseline HRR and death during hospitalization was established using restricted cubic spline analysis. We observed that the association between the HRR level and in-hospital mortality exhibited a non-linear curve (Figure 3). In the threshold analysis, we used a two-piecewise model to fit the link between the baseline HRR level and in-hospital mortality. We found an inflection point at 9.50 (Table 3). On the left side of the inflection point, the HR of HRR was 0.79 (95% CI: 0.70–0.90, *p* = 0.0003). This meant that the risk of in-hospital mortality was reduced by 21% per 1 unit increase. On the right side of the inflection point, the HR was 1.18 (95% CI: 0.91–1.53, *p* = 0.2158). It suggested that the association between HRR and in-hospital mortality was not statistically significant when the level of HRR was more than 9.50. This meant that the risk of in-hospital no longer decreased with increasing HRR. In our study, the *p*-value for the log-likelihood ratio test was 0.025 (Table 3).

**Table 3:** Threshold-effect analysis of the relationship between the baseline HRR level and in-hospital mortality in non-traumatic SAH patients.

**Models**	**Per-unit increase**		
	**HR**	**95%CI**	***p*-value**
**Model I**			
One line effect	0.88	0.80–0.96	0.0066
**Model II**			
Turning point (K)	9.50		
Baseline HRR levels < K	0.79	0.70–0.90	0.0003
Baseline HRR levels > K	1.18	0.91–1.53	0.2158
*p-*value for LRT test^*^			0.025

Model I, one-line linear regression model; Model II, two-piece wise linear regression model. Adjusted for age, sex, ethnicity, heart rate, RR, WBC, PT, APTT, congestive heart failure, Charlson comorbidity index, endovascular therapy, sepsis, Norepinephrine, vasopressin, ventilation, GCS, WFNS, delayed cerebral ischemia, hydrocephalus.

HR, Hazard Ratio; CI, confidence interval; LRT, logarithm likelihood ratio test.

^*^p < 0.05 indicates that Model II is significantly different from Model I.

### Subgroup analysis

**Figure 4:**
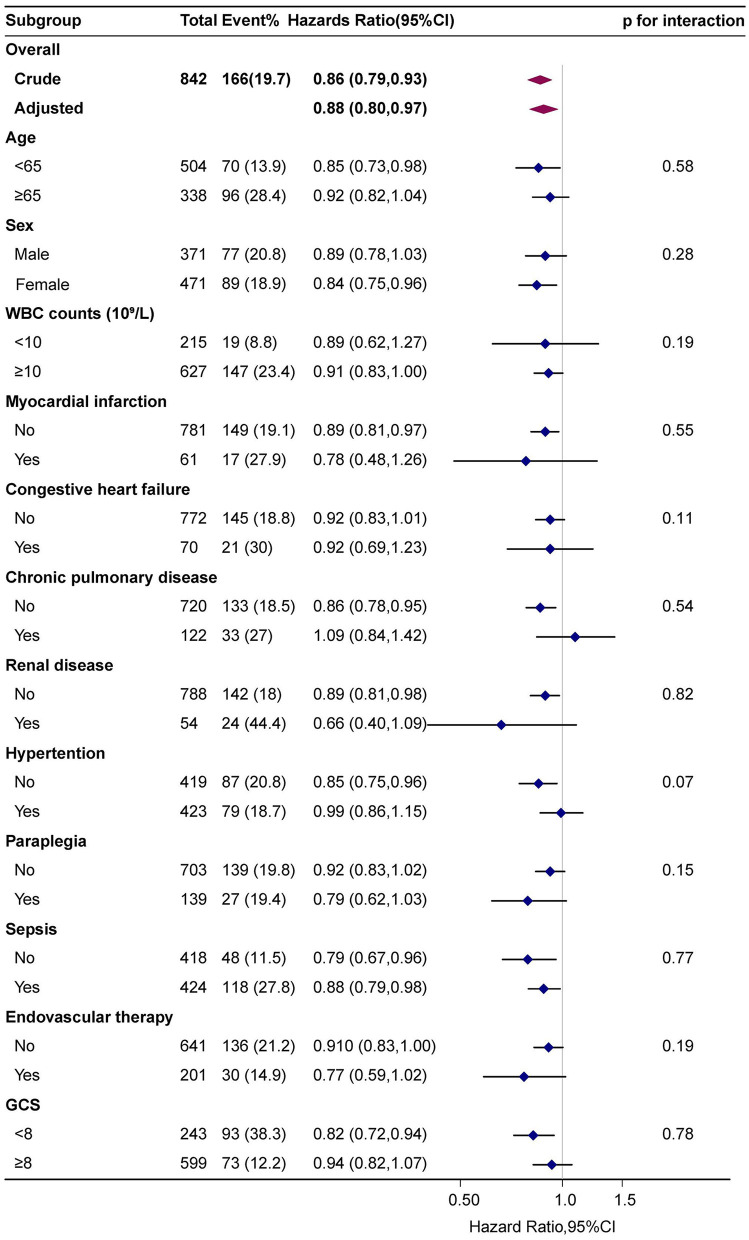
Subgroup analyses of the effect of hospital mortality. Adjusted for age, sex, ethnicity, HR, RR, WBC, PT, APTT, congestive heart failure, Charlson comorbidity index, endovascular therapy, sepsis, norepinephrine, vasopressin, ventilation, GCS, WFNS, except the subgroup variable.

The subgroup analysis was conducted to reveal the correlation between HRR and in-hospital mortality across age (< 65 and ≥65 years old), sex, WBC counts, myocardial infarction, congestive heart failure, chronic pulmonary disease, renal disease, paraplegia, hypertension, sepsis, endovascular therapy of aneurysm, GCS (< 8 and ≥8), and the results are shown in Figure 4. The interaction between the HRR and all subgroup factors was analyzed, and significant interactions were not observed (*p* for interaction > 0.05).

## Discussion

In the retrospective observational study, we investigated the relationship between the baseline HRR level and in-hospital mortality among non-traumatic SAH patients and made several important findings. First, we demonstrated that HRR was inversely associated with in-hospital mortality after adjusting for possible confounding factors. Second, we also found a threshold effect. When the HRR was < 9.50, the risk of in-hospital mortality decreased with the increase in HRR level. However, patients with progressively higher HRRs above this level did not show any further trend of mortality decreasing. Third, we observed no obvious interaction between the baseline HRR and in-hospital mortality, which indicated that HRR was independently associated with in-hospital mortality in different subgroups, even considering the surgical requirements.

HRR is easily obtained from a routine laboratory database without any external technology or cost and is significantly related to the level of inflammatory reactions (25–27). The HRR itself is not associated with mortality but rather the degree of inflammation that it represents. To the best of our knowledge, the relationship between the level of inflammation and mortality rate has long been a concern of non-traumatic SAH patients. The relationship between HRR and in-hospital mortality remains unknown, which prompted us to carry out the current study. A series of previous studies have demonstrated that a low level of HRR was associated with poor outcomes among multiple malignant diseases (25, 33, 38–41). In recent years, there was mounting evidence that a lower level of HRR was strongly associated with significantly worse outcomes in many critically ill patients, such as coronary heart disease (28), sepsis (29), ischemic stroke (30), and so on. One study with 6,046 participants found that a lower HRR (HRR < 10.25) in patients with percutaneous coronary atherosclerotic heart disease was associated with a 1.47-fold increased risk of long-term all-cause mortality. Qu et al. revealed an inverse association between lower HRR (< 9.76) and the risk of frailty in elderly patients with coronary heart disease, and HRR was identified as a stronger predictor of frailty than RDW or Hb. In our previous survey, a lower HRR (< 5.877) was also observed to be strongly associated with an increased risk of all-cause mortality in individuals with sepsis (29). Moreover, Qin et al. used the MIMIC-IV to determine the relationship between HRR and all-cause mortality in ischemic stroke patients and found that a lower HRR was associated with increased mortality in these patients (30). All the above results indicated that a lower HRR was associated with worse outcomes than a higher HRR. According to the findings above, we hypothesize that low HRR levels may increase the risk of mortality in patients with non-traumatic SAH. More experiments are needed to validate our hypothesis.

Several hypothesized mechanisms have been proposed to explain the reason why lower HRR leads to adverse outcomes in non-traumatic SAH patients. First, higher RDW in the normal range may mean red blood cell disruption or ineffective erythropoiesis (42). Furthermore, a higher RDW level also reflects an underlying inflammatory state and is related to adverse outcomes (43, 44). Forhecz et al. performed a retrospective cohort study involving 195 patients suffering chronic heart failure and found the correlation between RDW and inflammatory markers such as C-reactive protein, interleukin-6, soluble tumor necrosis factor (TNF) receptor I and II (45). Inflammation reactions will damage iron metabolism and inhibit the secretion of erythropoietin, resulting in a decrease in the hemoglobin level (46). In addition, after SAH, it usually triggers a physiological stress response, and increased release of endogenous catecholamine and cortisol, which may lead to secondary brain injury and potential inflammatory complications. Second, the oxygen-carrying capacity mainly depends on the hemoglobin level. The decrease in the hemoglobin value indicates that the oxygen supply of cerebral vessels is significantly reduced, and the oxygen supply of brain tissue is limited, which may lead to vasospasm. Moreover, extravasated red blood cells in the subarachnoid space undergo degradation, releasing a host of bioactive and potentially toxic molecules including hemoglobin, methemoglobin, and bilirubin, which have long been associated with the development of cerebral vasospasm and outcome (7, 8). Vasospasm will seriously affect the prognosis of patients with SAH (11, 12). Third, previous studies have shown that HRR was associated with the risk of frailty risk (26). Dysphagia, systemic infection, or anemia were the main causes of frailty in the early stage of SAH, which may aggravate the condition. Among individuals with lower HRR levels, we consistently noted a higher incidence of sepsis, which also explains this.

Our research has the following strengths: (1) This is the first study to examine the association between baseline HRR levels and in-hospital mortality in participants with non-traumatic SAH. (2) The study used real-world data to design a large-scale and diverse population study. The missing value of HRR was lower, which may reduce the selection bias. (3) We used a 2-piecewise Cox proportional risk regression model to analyze the threshold effect of the relationship between HRR and all-cause mortality. In addition, our findings may help clinicians identify high-risk participants with non-traumatic SAH.

However, the study has some limitations. First, due to its retrospective observational design, and thus, we were only able to provide the association between HRR and hospital mortality, and it is difficult to distinguish the cause and effect. Second, this single-center cohort may not fully represent the general patient population with non-traumatic SAH. Third, due to the limitations of the MIMIC database, missing information that could have affected the model was not collected, such as medications and acute stress. However, it should be noted that the potential results from these variables would bias toward the null, resulting in an undervaluation of the connection between HRR levels and hospital mortality. Four, we analyzed the first HRR record collected during ICU admission, and therefore, the results are limited to a confined period during which HRR was measured. We only collected data on hospitalization, so we only evaluated short-term results. Long-term results should be evaluated through further research. Nevertheless, the relationship between low HRR levels and hospital mortality was revealed.

## Conclusions

Therefore, patients with low HRRs should be given more attention, and their in-hospital mortality rate may be higher. This would benefit clinicians and contribute to better decisions.

## Data availability statement

The data analyzed in this study was obtained from the Medical Information Mart for Intensive Care IV (MIMIC-IV) database, the following licenses/restrictions apply: To access the files, users must be credentialed users, complete the required training (CITI Data or Specimens Only Research) and sign the data use agreement for the project. Requests to access these datasets should be directed to Physionet, https://doi.org/10.13026/7vcr-e114.

## Ethics statement

The studies involving human participants were examined and approved by Beth Israel Deaconess Medical Center. To protect patient privacy, all data were de-identified; therefore, the Ethical Committee of the Beth Israel Deaconess Medical Center waived the requirement for informed consent. Written informed consent for participation was not required for this study in accordance with the national legislation and the institutional requirements.

## Author contributions

Conceptualization, methodology, software, validation, formal analysis, investigation, resources, data curation, writing—original draft preparation, writing—review and editing, visualization, and supervision: JW and JL. All authors contributed to the article and approved the submitted version.
