# Supplementary material for: Association between hemoglobin-to-red blood cell distribution width ratio and hospital mortality in patients with non-traumatic subarachnoid hemorrhage
Source: Front Neurol. 2023 Jun 14;14:1180912. doi: 10.3389/fneur.2023.1180912 (PMC10303799; doi:10.3389/fneur.2023.1180912)
Supplement: Supplementary file 1 [file Data_Sheet_1.PDF]

Supplementary Table 1 | Details of missing values.

Variable	The number of missing values	The percent of missing values (%)
Temperature	6	0.7126
Spo2	1	0.1188
SBP	4	0.4751
RR	2	0.2375
PT	10	1.1876
MBP	1	0.1188
HR	1	0.1188
DBP	4	0.4751
APTT	14	1.6627

Abbreviations: HR, heart rate; SBP, systolic blood pressure; DBP, diastolic blood pressure; MBP, mean blood pressure; RR, respiratory rate; SpO2, percutaneous oxygen saturation; PT, prothrombin time; APTT, activated partial thromboplastin time;

Supplementary Table 2. The baseline clinical characteristics of patients with non-traumatic SAH.

Variables	Total (n = 842)	Survival (n = 676)	Non-Survival (n = 166)	*p*-Value
Demographic				
Female, n (%)	471 (55.9)	382 (56.5)	89 (53.6)	0.501
Age, years	61.2 ± 14.9	59.8 ± 14.5	66.8 ± 14.9	< 0.001
Ethnicity, n (%)				< 0.001
Asian	32 (3.8)	23 (3.4)	9 (5.4)	
White	507 (60.2)	439 (64.9)	68 (41)	
Black	68 (8.1)	57 (8.4)	11 (6.6)	
Other	235 (27.9)	157 (23.2)	78 (47)	
Vital signs				
HR, beats/minute	77.0 (70.0, 87.0)	76.0 (69.0, 85.2)	82.0 (75.0, 92.0)	< 0.001
SBP, mmHg	125.3 ± 12.9	125.5 ± 12.6	124.6 ± 14.2	0.437
DBP, mmHg	64.3 ± 8.9	64.4 ± 8.8	63.7 ± 9.4	0.328
MBP, mmHg	82.5 ± 8.6	82.6 ± 8.6	82.1 ± 8.4	0.458
RR, times/minute	18.0 (16.0, 20.0)	17.0 (16.0, 19.0)	19.0 (17.0, 21.8)	< 0.001
Temperature, °C	37.0 (36.8, 37.3)	37.0 (36.8, 37.3)	37.1 (36.6, 37.5)	0.325
SpO2, %	98.0 (96.0, 99.0)	97.5 (96.0, 99.0)	98.0 (97.0, 99.0)	0.001
Comorbidities, n (%)				
Myocardial infarction	61 (7.2)	44 (6.5)	17 (10.2)	0.097
Congestive heart failure	70 (8.3)	49 (7.2)	21 (12.7)	0.024
Chronic pulmonary disease	122 (14.5)	89 (13.2)	33 (19.9)	0.028
Paraplegia	139 (16.5)	112 (16.6)	27 (16.3)	0.925
Renal disease	54 (6.4)	30 (4.4)	24 (14.5)	< 0.001
Hypertension	423 (50.2)	344 (50.9)	79 (47.6)	0.446
Diabetes	39 (4.6)	29 (4.3)	10 (6)	0.341
Sepsis	424 (50.4)	306 (45.3)	118 (71.1)	< 0.001
Charlson comorbidity index	4.0 (3.0, 6.0)	4.0 (3.0, 6.0)	5.0 (4.0, 7.0)	< 0.001
Laboratory results				
Hemoglobin, g/L	12.2 (11.0, 13.4)	12.3 (11.2, 13.5)	11.8 (10.3, 13.3)	0.016
RDW, %	13.4 (12.9, 14.3)	13.3 (12.8, 14.0)	14.1 (13.3, 15.4)	< 0.001
HRR	9.2 (7.9, 10.2)	9.3 (8.1, 10.2)	8.5 (6.9, 9.8)	< 0.001
Platelets, 10^9/L	230.0 (187.0, 280.8)	232.5 (190.0, 281.0)	221.5 (157.0, 279.8)	0.011
WBC, 10^9/L	12.9 (9.9, 16.5)	12.5 (9.5, 15.7)	15.4 (11.9, 19.7)	< 0.001
Calcium, mg/dl	8.7 (8.3, 9.1)	8.7 (8.4, 9.1)	8.8 (8.3, 9.3)	0.438
MCH, pg	30.5 (29.2, 31.8)	30.4 (29.2, 31.8)	30.6 (28.9, 31.9)	0.984
PT, s	12.6 (11.7, 13.8)	12.4 (11.7, 13.5)	13.6 (12.3, 15.7)	< 0.001
APTT, s	28.8 (25.9, 33.1)	28.5 (25.9, 32.5)	29.9 (26.7, 37.1)	0.014
Glucose, mg/dl	130.9 (113.4, 153.5)	128.0 (111.2, 148.8)	149.8 (125.1, 183.3)	< 0.001
Therapy, n (%)				
Norepinephrine	30 (3.6)	13 (1.9)	17 (10.2)	< 0.001
Vasopressin	8 (1.0)	2 (0.3)	6 (3.6)	0.001
Ventilation	429 (51.0)	276 (40.8)	153 (92.2)	< 0.001
Endovascular therapy of aneurysm	201 (23.9)	171 (25.3)	30 (18.1)	0.05
Clipping of aneurysm	35 (4.2)	30 (4.4)	5 (3)	0.41
Scores				
GCS	12.0 (7.0, 14.0)	13.0 (8.0, 14.0)	6.0 (3.0, 15.0)	< 0.001
WFNS				< 0.001

I	127 (15.1)	80 (11.8)	47 (28.3)	
II	172 (20.4)	171 (25.3)	1 (0.6)	
III	115 (13.7)	111 (16.4)	4 (2.4)	
IV	185 (22.0)	164 (24.3)	21 (12.7)	
V	243 (28.9)	150 (22.2)	93 (56)	
Outcomes				
Length of ICU stay, days	7.0 (2.9, 12.9)	7.4 (3.4, 13.5)	3.9 (2.0, 9.4)	< 0.001
Length of hospital stay, days	11.3 (6.7, 18.7)	12.1 (7.9, 20.3)	4.2 (1.8, 11.4)	< 0.001

Abbreviations: HR, heart rate; SBP, systolic blood pressure; DBP, diastolic blood pressure; MBP, mean blood pressure; RR, respiratory rate; SpO2, percutaneous oxygen saturation; RDW, red cell distribution width; HRR, hemoglobin/red cell distribution width; WBC, white blood cell; MCH, mean corpuscular hemoglobin; PT, prothrombin time; APTT, activated partial thromboplastin time; GCS, Glasgow coma score; WFNS, World Federation of Neurosurgical Societies; ICU, Intensive care unit; SAH, subarachnoid hemorrhage.

Supplementary Table 3| Univariate Cox regression analyses for in-hospital mortality in patients with non-traumatic SAH.

Variable	HR 95 CI%	P value
Gender	0.978 (0.719,1.331)	0.8899
Age	1.028 (1.017,1.039)	< 0.001
Ethnicity		
White	0.471 (0.235,0.947)	0.0345
Black	0.507 (0.205,1.258)	0.143
Other	1.161 (0.582,2.319)	0.6714
HR	1.022 (1.01,1.033)	< 0.001
SBP	0.9939 (0.982,1.006)	0.3218
DBP	0.9926 (0.9753,1.0102)	0.4088
MBP	0.9902 (0.9726,1.0082)	0.2827
RR	1.13 (1.085,1.176)	< 0.001
Temperature	0.839 (0.622,1.131)	0.2492
Spo2	0.988 (0.907,1.077)	0.7844
Glucose	1 (0.9999,1.0001)	0.7441
Platelet	0.9983 (0.9964,1.0001)	0.069
WBC	1.044 (1.027,1.06)	< 0.001
Calcium	1.246 (1,1.553)	0.0502
MCH	0.976 (0.919,1.037)	0.4335
PT	1.012 (1.002,1.021)	0.0152
APTT	1.0053 (1.0005,1.0101)	0.0303
Myocardial infarction	1.375 (0.832,2.272)	0.214
Congestive heart failure	1.358 (0.855,2.155)	0.1947
Dementia	0.895 (0.285,2.814)	0.8501
Paraplegia	0.874 (0.578,1.323)	0.5245
Chronic pulmonary disease	1.379 (0.94,2.022)	0.1002
Hypertension	0.875 (0.644,1.189)	0.3922
Diabetes	1.324 (0.698,2.51)	0.3904
Sepsis	1.578 (1.115,2.235)	0.0101
Charlson comorbidity index	1.152 (1.088,1.219)	< 0.001
Endovascular therapy of aneurysm	0.591 (0.397,0.878)	0.0093
Clipping of aneurysm	0.598 (0.245,1.458)	0.2584
Norepinephrine	2.62 (1.567,4.381)	< 0.001

Vasopressin	5.051 (2.232,11.43)	< 0.001
Ventilation	9.213 (5.209,16.294)	< 0.001
GCS	0.907 (0.873,0.943)	< 0.001
WFNS		
Ⅱ	0.01 (0.001,0.072)	< 0.001
Ⅲ	0.053 (0.019,0.149)	< 0.001
Ⅳ	0.138 (0.082,0.235)	< 0.001
Ⅴ	0.466 (0.32,0.679)	< 0.001
Length of hospital stay	0.007 (0.003,0.015)	< 0.001
Length of ICU stay	0.881 (0.855,0.908)	< 0.001

Abbreviations: HR, heart rate; SBP, systolic blood pressure; DBP, diastolic blood pressure; MBP, mean blood pressure; RR, respiratory rate; SpO2, percutaneous oxygen saturation; RDW, red cell distribution width; WBC, white blood cell; MCH, mean corpuscular hemoglobin; PT, prothrombin time; APTT, activated partial thromboplastin time; GCS, Glasgow coma score; WFNS, World Federation of Neurosurgical Societies; ICU, Intensive care unit; SAH, subarachnoid hemorrhage.
